# The impact of ICAM-1, CCL2 and TGM2 gene polymorphisms on differentiation syndrome in acute promyelocytic leukemia

**DOI:** 10.1186/s12885-021-07783-y

**Published:** 2021-01-09

**Authors:** Zahra Mohammadzadeh, Azadeh Omidkhoda, Bahram Chahardouli, Ghazaleh Hoseinzadeh, Kamran Ali Moghaddam, Seyed Asadollah Mousavi, Shahrbano Rostami

**Affiliations:** 1 Hematology and Blood Banking Department, School of Allied Medical Sciences, Tehran University of Medical Sciences, Tehran, Iran; 2 Hematology-Oncology and Stem Cell Transplantation Research Center, Tehran University of Medical Sciences, Tehran, Iran

**Keywords:** Acute Promyelocytic leukemia, Differentiation syndrome, ICAM-1, CCL2 chemokine, Transglutaminase II, SNPs

## Abstract

**Background:**

Although arsenic trioxide (ATO) and all-trans retinoic acid (ATRA) are well-tolerated and effective treatments for Acute Promyelocytic Leukemia (APL), Differentiation Syndrome (DS) is a lethal side effect in some patients. The pathogenesis of DS is complex and not well understood; however, it is considered as an inflammatory response due to cytokines release of differentiated cells. Moreover, adhesion molecules that are widely expressed on the surface of differentiated cells and gene expression changes of transglutaminase2 (TGM2) are mechanisms involved in the development of DS. The purpose of this study was to assess the association of single nucleotide polymorphisms (SNP) of Intercellular Adhesion Molecule-1 (ICAM-1), chemokine (C-C motif) ligand 2 (CCL2) and TGM2 as inflammatory factors with differentiation syndrome susceptibility.

**Methods:**

DNA was extracted from 133 APL patients and 100 normal controls. Assessment according to the PETHEMA criteria revealed that 13.5% of these patients experienced differentiation syndrome. Tetra-ARMS PCR and PCR-RFLP were done to amplify DNA fragments in APL patients with and without DS. Then DNA sequencing was done to validate the results. SNPStats, SPSS and Finch TV were used to analyze the results.

**Results:**

A significant correlation was found between rs4811528 in the TGM2 gene and differentiation syndrome susceptibility (*P* = 0.002, 95% CI = 1.74–18.81, OR = 5.72) while rs5498 in ICAM-1, rs1024611 in CCL2, and rs7270785 in TGM2 genes showed no correlation with differentiation syndrome. The G allele of rs7270785 and rs4811528 showed a haplotypic association with differentiation syndrome (*P* = 0.03, 95% CI = 1.13–13.86, OR = 3.96).

**Conclusions:**

AA genotype of the *TGM2* SNP (rs4811528) may be a risk factor for development of DS in patients with APL following the use of ATRA/ATO.

## Background

Acute Promyelocytic Leukemia (APL) is characterized by a reciprocal translocation between the long arms of chromosomes 15 and 17, t (15; 17) (q21; q22), which results in a fusion between the promyelocytic leukemia (PML) gene and retinoic acid receptor alpha (RAR alpha). PML/RARα homodimers inhibit the expression of differentiation genes in granulocytes [1, 2]. All-trans retinoic acid (ATRA) and arsenic trioxide (ATO), especially when combined compared to either one alone, are the most effective drugs for the treatment of APL, which induce the degradation of chimeric oncoprotein PML/RARα and APL cell differentiation [3, 4]. ATRA/ATO treatment can induce differentiation syndrome (DS) or retinoic acid syndrome (a life-threatening complication) in some patients [5]. According to the PETHEMA (Programa para el Tratamiento de Hemopatías Malignas) criteria, the presence of one or more of the following features may indicate a diagnosis of DS: hyperleucocytosis, respiratory distress, unexplained fever, hypotension, weight gain more than 5 kg, acute renal failure, and a chest radiograph demonstrating pericardial effusion or pulmonary infiltrates [6–9]. The molecular and cellular mechanisms of DS are not fully known; however, it is believed that administration of ATRA/ATO leads to a systemic inflammatory response syndrome (SIRS) and massive gene expression changes in differentiating blast cells [9–11]. The proposed mechanisms include changes in the adhesion molecules, cytokine secretion, and enzymes during ATRA/ATO induced differentiation such as Intercellular Adhesion Molecule-1(ICAM-1), monocyte chemoattractant protein-1 (MCP-1/CCL2), and type-2 transglutaminase (TGM2/TG2) [10, 12–14]. ICAM-1 (CD54) is a single chain 76–110 kDa glycoprotein and a member of the Ig superfamily located on chromosome 19p13 [15], MCP-1/CCL2 is a CC chemokine located on chromosome 17q11 [16], and TGM2 is a 74–80 kDa protein and a member of the transglutaminase family located on chromosome 20q11–12 [17, 18]. Single Nucleotide Polymorphisms (SNPs) SNPs are variations in the DNA sequence. SNPs are helpful in determining how individuals respond to diseases or interact with drugs and therapeutic procedures. Many studies have shown associations between polymorphisms and inflammatory disorders [19–21]. One study found an association between the AA genotype at *ICAM-1* Exon 6 (E469K) and DS [3]. Considering the possible role of the polymorphisms of cell adhesion molecules, chemokines, and transglutaminase in DS pathogenesis, the aim of this study was to investigate the association of rs1024611 in CCL2, rs5498 in *ICAM-1*, and rs7270785 and rs4811528 in *TGM2* with the development of differentiation syndrome in patients treated with ATRA/ATO.

## Methods

### Patients characteristics

From 2012 to 2017, patients with APL who referred to the Hematology, Oncology, and Stem Cell Transplantation Research Center of Shariati hospital, Tehran, Iran were studied. All patients received ATRA-ATO as reported previously [22]. One hundred and thirty-three APL patients were selected based on availability of patient samples. According to the PETEHMA criteria (fever ≥38 °C, weight gain> 5 kg, hypotension, dyspnea, LQTS (Long QT Syndrome) and acute renal failure), Eighteen selected APL patients were diagnosed with differentiation syndrome after receiving ATRA/ATO. Samples from 100 healthy volunteers were used as controls. The study was approved by the Ethics Committee of Tehran University of Medical Sciences (Ethics Code: IR.TUMS.SPH.REC.1397.269) and written informed consent was obtained from all participants.

### DNA isolation

The genomic DNA of the samples was extracted from their peripheral blood in tubes containing ethylene-diamine tetra acetic acid (EDTA) anticoagulants using the standard salting-out method. The concentration and the purity of the DNA samples were evaluated with a Nano Drop device (Thermo Fisher Scientific, USA).

### Tetra-ARMS PCR (tetra-primer ARMS PCR)

The amplification-refractory mutation system polymerase chain reaction (Tetra-ARMS PCR) was used for detection of rs5498 in *ICAM-1* and rs7270785 and rs4811528 in *TGM2* with appropriate primer sets Table 1. The reaction was performed in a total volume of 15 μl, containing 1 μl genomic DNA (60–80 ng/μl), 7.5 μl 1× Master Mix PCR (Ampliqon, Denmark), optimum forward and reverse inner primer ratio for rs5498, rs4811528 and rs7270785 (1:2, 1:2 and 1:4, respectively (10 pmol)), and 0.2–0.5 μM of each outer primer. The optimum PCR condition was 95 °C for 3 min followed by 35 cycles (95 °C for 15 s, 62 °C for 20 s, and 72 °C for 20 s in rs5498; 95 °C for 15 s, 62 °C for 15 s, and 72 °C for 25 s for rs4811528; and 95 °C for 15 s, 68 °C for 20 s, and 72 °C for 20 s for rs7270785) and a final extension at 72 °C for 6 min. To visualize the results, 10 μl PCR product was run on a 2% agarose gel containing 3 μl loading dye.

**Table 1:** The primer sequences used for gene PCR

SNP ID	Primer sequences		Product size (bp)
**rs5498**	F inner	GAGCACTCAAGGGGAGGTCACCCTCG	G allele (189)
	R inner	TCACTCACAGAGCACATTCACGGTCACATT	A allele (274)
	F outer	ATCTCATCGTGTTTTTCCAGATGGCCCC	Control band (407)
	R outer	CCCATTATGACTGCGGCTGCTACCACA	
**rs4811528**	F inner	ATAAACCTTGGCAAGCTCAAGGTCAGGGTT	A allele (179)
	R inner	CACTCCTCCCACCTTAAGGGCTTCTCC	G allele (259)
	F outer	GCTGTGTTGCTGTGTGAGCCTGGATAAG	Control band (383)
	R outer	TGGAATAGTCGATGGTGAGCAGGAGACC	
**rs7270785**	F inner	CTTATCTCAAACCATAACCAACCTGCACC	T allele (201)
	R inner	CAAGCTACAATGTTCCCACACAGGAGCA	G allele (291)
	F outer	CAAGCTACAATGTTCCCACACAGGAGCA	Control band (435)
	R outer	CTTCTCCAATTGTCTGGGCAGCGTAGTG	
**rs1024611**	F outer	GGCTGAGTGTTCACATAGGCTTCTGAGT	Control band (281)
	R outer	AACTTCCAAAGCTGCCTCCTCAGAGT	

*F* Forward, *R* Reverse

### PCR-RFLP

The *CCL2* polymorphism (rs1024611) was detected by polymerase chain reaction-restriction fragment length polymorphism (PCR-RFLP) using forward and reverse primers (Table 1). The reaction was performed in a total volume of 15 μl, containing 1 μl genomic DNA (60–80 ng/μl), 7.5 μl 1× Master Mix PCR (Ampliqon, Denmark), and 0.3 μM of each outer primer. PCR was performed by denaturing the samples at 95 °C for 3 min followed by 40 cycles including 95 °C for 15 s, 64 °C for 20 s, 72 °C for 25 s, and final extension at 72 °C for 6 min. For RFLP analysis, the PCR product was digested with 10 U/ μl PVUП and 10 x buffer G (Thermo Scientific, Massachusetts, United States) and incubated at 37 °C for 16 h. The digested PCR products were separated on a 2% agarose gel.

### Validation assay

DNA sequencing was done to validate the results. The same outer primers of Tetra-ARMS PCR and PCR-RFLP in conventional PCR were used to amplify the regions containing SNPs. Then, the cycle sequencing reaction was done using the Big Dye Terminator v3.1 Cycle Sequencing kit (Applied Biosystems, Foster City, CA, USA) according to the manufacturer’s instructions and the samples were sequenced using the 3130 xl Genetic Analyzer ABI.

### Statistical analysis

The Hardy-Weinberg equilibrium (HWE) was applied to assess the deviation of the genotype or allele frequency. The demographic and hematologic data were distributed normally. Pearson’s Chi square, Mann–Whitney U-test, and t-test were used for statistical comparison. The SPSS version 20.0 (IBM, NY, USA) was used for data analysis. Multiple logistic regression models (codominant, dominant, recessive, over dominant and log-additive) were applied to analyze the correlation between the SNP data and disease phenotype (odds ratio) with 95% confidence interval was calculated using the SNPStats software. *P* values less than 0.05 were considered significant. The Finch TV software version 1.4.0 was used to interpret the sequencing results.

## Results

### Patients ‘baseline characteristics

One hundred and thirty-three APL patients (74 females and 59 males) with a mean age of 36 ± 13.9 years and 100 healthy volunteers (51 females and 49 males) with a mean age of 34 ± 13.5 years were included in the study*.*

There were no significant differences between APL patients and healthy controls (*p* value < 0.05). Table 2 presents demographics and laboratorial findings of APL patients, with and without DS. According to this table, no significant differences were detected between the two groups.

**Table 2:** Demographics and laboratorial findings of APL patients with and without DS

Characteristics	DS	No DS	***P*** value
Number of samples	18	115	
Gender (Male/Female)	7/11	52/63	0.6
Age (years)	37.8 ± 15.8	34.6 ± 13	0.4
WBC counts (/μl*10^3^)	61.3 ± 9.9	65.5 ± 12.2	0.4
Hb (g/dl)	1.7 ± 8	2.6 ± 9	0.1
Platelets (/μl*10^3^)	53.4 ± 45	57.2 ± 76	0.2

Values presented as mean (±SD), *Hb* Hemoglobin

### Electrophoresis and sequencing results

**Fig. 1:**
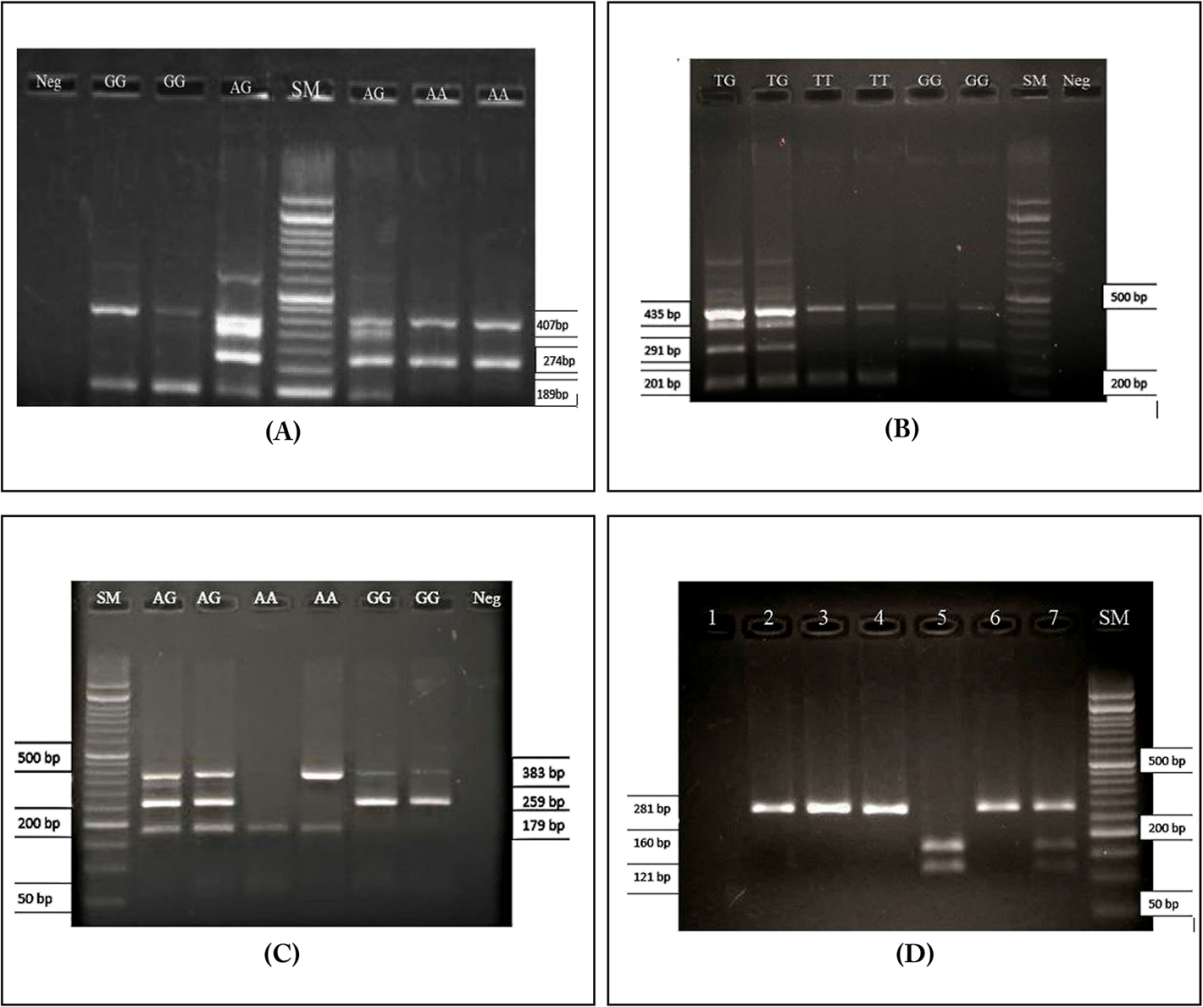
Agarose gel electrophoresis of genotype variation of rs5498, rs7270785, rs4811528 and PCR-RFLP for rs1024611. Genotype variation of rs5498 determined by control fragment (407 bp), specific fragment of A allele (274 bp) and G allele (189 bp) **a**. Genotype variation of rs7270785 determined by control fragment (435 bp), specific fragment of G allele (291 bp) and T allele (201 bp) **b**. Genotype variation of rs4811528 determined by control fragment (383 bp), specific fragment of G allele (259 bp) and A allele (179 bp) **c**. For rs1024611, 1 represent negative control; 2 (undigested) and 3 (digested) represent T allele (281 bp);4 (undigested) and 5 (digested) represent C allele (160 bp and 121 bp); 6 (undigested) and 7 (digested) represent T and C allele (281 bp, 160 bp and 121 bp) **d**

The product of the tetra-primer ARMS PCR specific for rs5498 contained 3 fragments (407 bp, 274 bp and 189 bp) in the AG heterozygous genotype and 2 fragments in the homozygous mutant and wild types (GG resulting in 189 bp and 407 bp fragments, and AA resulting in 274 bp and 407 bp fragments) Fig. 1a**.** In rs7270785, the TG heterozygous genotype contained 3 fragments (435 bp, 291 bp and 201 bp), the GG homozygous mutant genotype contained 2 fragments (435 bp and 291 bp), and the TT homozygous wild type contained 2 fragments (435 bp and 201 bp) Fig. 1b. In rs4811528, the AG heterozygous genotype had 3 fragments (383 bp, 259 bp and 179 bp), the GG mutant homozygous genotype had 2 fragments (383 bp and 259 bp), and the AA homozygous wild type had 2 fragments (383 bp and 179 bp) Fig. 1c**.** For rs1024611, the amplified fragment length was 281 bp. Digestion with PVUП produced 3 fragments of 281 bp, 160 bp and 121 bp in the CT heterozygous genotype and 2 fragments of 160 bp and 121 bp in the CC homozygous mutant genotype while the TT homozygous wild type remained uncut Fig. 1d**.** A heterozygous sample of each SNP was selected that contained both mutant and wild type alleles; then, DNA sequencing confirmed the results obtained by T-ARMS PCR.

### Distribution of genotype and allele frequency

The genotype and allele frequencies for rs5498, rs4811528, rs7270785, and rs1024611 polymorphisms in case and control groups are presented in Table 3. The distribution of genotypes was consistent with HWE in both groups (*P* > 0.05). The frequency of the genotypes of *TGM2* gene polymorphism (rs4811528) was AG = 44%, AA = 37%, and GG = 19% in 133 patients with APL and AA = 48%, AG = 41%, and GG = 11% in 100 controls.

**Table 3:** Genotypes and allele frequencies of polymorphisms analyzed in APL Patients and controls

	APL Patients				controls			
**SNP ID**	**Genotype (*N*)**	**Genotype Frequency (%)**	**Allele (*N*)**	**Allele Frequency (%)**	**Genotype**	**Genotype Frequency (%)**	**Allele (*N*)**	**Allele Frequency (%)**
**rs5498**	AA (39)	29	A (150)	56	AA	30	A (112)	56
	AG (72)	54	G (116)	44	AG	52	G (88)	44
	GG (22)	17			GG	18		
**rs4811528**	AA(50)	37	A (158)	59	AA	48	A (137)	68
	AG (58)	44	G (108)	41	AG	41	G (63)	32
	GG (25)	19			GG	11		
**rs7270785**	TT (38)	29	T (148)	56	TT	25	T (108)	54
	TG (72)	54	G (118)	44	TG	58	G (92)	46
	GG (23)	17			GG	17		
**rs1024611**	TT (61)	46	T (181)	68	TT	54	T (144)	72
	TC (59)	44	C (85)	32	TC	36	C (56)	28
	CC (13)	10			CC	10		

### Correlation of ICAM-1, CCL2 and TGM2 polymorphisms with differentiation syndrome in APL patients

Five genetic models (codominant, dominant, recessive, over dominant and log-additive) were applied to analyze the associations between *ICAM-1, CCL2* and *TGM2* polymorphisms and differentiation syndrome in patients with APL. The results of the genetic models showed that TGM2 polymorphism (rs4811528) was significantly associated with susceptibility to differentiation syndrome [codominant (OR = 5.61; 95% CI = 1.53–20.48, *P* = 0.009), dominant (OR = 5.72; 95% CI = 1.74–18.81, *P* = 0.002), over dominant (OR = 4.06; 95% CI = 1.14–14.44, *P* = 0.02), and log-additive (OR = 3.78; 95% CI = 1.37–10.44, *P* = 0.004)] Table 4. There was no significant association between *ICAM-1* (rs5498), *CCL2* (rs1024611), and *TGM2* (rs7270785) polymorphisms and DS (data not shown). The 2-SNP haplotypes analysis also revealed a haplotypic association between the rs7270785–rs4811528 haplotypes of *TGM2* gene with DS development in G allele (OR = 3.96; 95% CI = 1.13–13.86, *P* = 0.033) Table 5.

**Table 4:** Analysis of association of TGM2 polymorphism (rs4811528) with the development of DS in APL patients

Model	Genotype	DS (%)	No DS (%)	OR (95% CI)	***P***-value	AIC	BIC
**Codominant**	A/A	13 (72.2)	37 (32.2)	1.00	0.0096	90.2	104.7
	A/G	4 (22.2)	54 (47)	5.61 (1.53–20.48)			
	G/G	1 (5.6)	24 (20.9)	6.14 (0.70–53.45)			
**Dominant**	A/A	13 (72.2)	37 (32.2)	1.00	0.0023	88.2	99.8
	A/G-G/G	5 (27.8)	78 (67.8)	5.72 (1.74–18.81)			
**Recessive**	A/A-A/G	17 (94.4)	91 (79.1)	1.00	0.24	96.2	107.7
	G/G	1 (5.6)	24 (20.9)	3.03 (0.36–25.25)			
**Over dominant**	A/A-G/G	14 (77.8)	61 (53)	1.00	0.02	92.1	103.6
	A/G	4 (22.2)	54 (47)	4.06 (1.14–14.44)			
**Log-additive**	–	–	–	3.78 (1.37–10.44)	0.004	89.2	100.8

*AIC* Akaike Information criterion, *BIC* Bayesian Information

**Table 5:** Analysis of Haplotypic effect of rs4811528 and rs7270785 with the development of DS in APL patients

rs7270785	rs4811528	Frequency	OR (95% CI)	***P***-value
T	A	0.43	1.00	0.033
G	G	0.27	3.96 (1.13–13.86)	
G	A	0.17	1.47 (0.55–3.95)	0.45
T	G	0.13	6.06 (0.67–54.75)	0.11

## Discussion

Differentiation syndrome (DS) is a life-threatening complication characterized by respiratory distress, unexplained fever, hypotension, weight gain, interstitial pulmonary infiltrates, pleural or pericardial effusion, and acute renal failure as described by Frankel et al. in 1992 [7, 23]. Although the pathogenesis of DS is complex and not well understood, several molecular and cellular mechanisms are involved in the development of DS. ATRA is thought to (a) lead to the release of a variety of cytokines (especially inflammatory cytokines) by differentiating blast cells and (b) induce changes in the adhesive properties of blasts cells as well as massive changes in gene expression, including downregulation of cell proliferation of related genes and induction of genes involved in immune function [9, 24]. Finally, ATRA induces a systemic inflammatory response syndrome (SIRS) that manifests as fever, tachycardia and tachypnea and can progress to shock if left untreated [9]. The incidence of DS in APL patients ranges from 2 to 27%, possibly due to the heterogeneity and range of clinical symptoms as well as inaccuracy of diagnostic criteria [25]. In the current study, DS was diagnosed in 18 of 133 APL patients (13.5%) according to the PETEHMA criteria, including fever (temperature ≥ 38 °C), weight gain> 5 kg, hypotension, dyspnea, long QT syndrome, and acute renal failure after taking ATRA/ATO. This is the first study of the role of several polymorphisms (*ICAM-1, CCL2* and *TGM2* genes) in the susceptibility of APL patients receiving ATO/ATRA to differentiation syndrome. MCP-1 regulates the migration of monocytes/macrophages to tissues and is required for response to inflammation and routine immunological surveillance of tissues [26]. An A to G single nucleotide polymorphism (SNP) in the *CCL2* enhancer region (rs1024611, originally designated as –2518G or –2578G) has been associated with several chronic inflammatory conditions such as systemic lupus erythematosus (SLE) and rheumatoid arthritis [26–28]. In the present study, no association was found between this polymorphism and the development of differentiation syndrome. ICAM-1 is involved in cell adhesion and signaling, plays an important role in tumor progression and tumorigenesis, specifically by facilitating tumor invasion, and is associated with susceptibility to many cancers, including acute promyelocytic leukemia (APL) [14, 15]. Dore et al. found an association between development of DS and the AA genotype at codon 469 of *ICAM-1* [3]. However, the present study showed no significant association between exon 6 (E469K) of *ICAM-1* polymorphism and DS. The inclusion and exclusion criteria of the patients with DS and the prescribed medicines were different between the present study and the study by Dore et al. Type-2 transglutaminase (TGM2/TG2) is emerging as a multifunctional enzyme that is capable of promoting specialized enzyme functions under regulation by allosteric effectors depending on its cellular location such as angiogenesis, cell growth/differentiation, and cell death [17, 18]. TGM2 acts as a G protein in signal transduction processes and is involved in the pathophysiology of various inflammatory conditions. TGM2 is associated with 329 diseases, including immune system, endocrine, metabolic, cardiovascular, epidermal, renal and hematological diseases [17]. Bradford et al. genotyped eight SNPs (rs2076380, rs7270785, rs4811528, rs2284879, rs6023526, rs2268909, rs17789815 and rs1555074) related to the *TGM2* gene in individuals with schizophrenia in a British population. The rs7270785–rs4811528 haplotypes showed the strongest association with schizophrenia, and the authors suggested that the *TGM2* gene might be involved in schizophrenia in the British population [12]. Wang et al. found no genetic association between four SNPs (rs2076380, rs7270785, rs4811528, and rs6023526) related to the *TGM2* gene and schizophrenia in a Chinese population [29]. Csomós et al. reported that TGM2 played an important role in neutrophil granulocyte differentiation and gene expression and argued that reduced expression of TGM2 in the NB4 model of acute promyelocytic leukemia might suppress effector functions of neutrophil granulocytes and attenuate the ATRA-induced inflammatory phenotype of DS [10]. According to Jambrovics et al., *TGM2* expression is induced by all-trans retinoic acid in differentiated NB4 cells and nuclear factor kappa-light-chain (NF-kB) signaling network is responsible for driving pathogenic processes by initiating an inflammatory cascade through over-expression of interleukin 1 beta (IL-1β), numerous cytokines, and tumor necrosis factor alpha (TNF-α) [30]. A limitation of the present study was that the number of patients with DS was relatively small and further studies in a bigger population with DS are necessary to confirm the findings.

## Conclusion

AA genotype of the *TGM2* SNP (rs4811528) may be a risk factor for development of DS in patients with APL following the use of ATRA/ATO.

## Data Availability

The datasets used and analyzed during the current study are available from the corresponding author on reasonable request.

## Abbreviations

APL: Acute Promyelocytic Leukemia
ATRA: All Trans Retinoic Acid
ATO: Arsenic Trioxide
SNP: Single Nucleotide Polymorphism
CCL2: CC motif Chemokine Ligand 2
ICAM-1: Intercellular Adhesion Molecule-1
DS: Differentiation Syndrome
PETHEMA: Programa para el Tratamiento de Hemopatías Malignas
PCR: Polymerase Chain Reaction
RFLP: Restriction Fragment Length Polymorphism
ARMS: The Amplification-refractory Mutation System
LQTS: Long QT Syndrome
TGM2: Transglutaminase 2
